# Parasitoids of the Sawfly, *Arge pullata*, in the Shennongjia National Nature Reserve

**DOI:** 10.1673/031.012.9701

**Published:** 2012-08-13

**Authors:** Tao Li, Mao-Ling Sheng, Shu-Ping Sun, You-Qing Luo

**Affiliations:** ^1^ The Key Laboratory for Silviculture and Conservation of Ministry of Education, Beijing Forestry University, Beijing 100083, P. R. China; ^2^ General Station of Forest Pest Management, State Forestry Administration, Shenyang Liaoning I 10034, P. R. China

**Keywords:** *Betula* spp., biological control, hyperparasitoid, parasitism rate, *Vibrissina turrita*

## Abstract

Larvae of the argid sawfly, *Arge pullata* (Zaddach) (Hymenoptera: Argidae), feeds on leaves of birch (*Betula* spp.) in China, Europe, Siberia, and Japan. Parasitoids of *A. pullata* were studied in Shennongjia National Nature Reserve, Hubei Province, China, in 2009 and 2010. Five parasitoid species were found: *Pleolophus suigensis* (Uchida), *Mastrus nigrus* Sheng, *Endasys parviventris nipponicus* (Uchida) (Hymenoptera: Ichneumonidae), *Vibrissina turrita* (Meigen) (Diptera: Tachinidae) and *Conura xanthostigma* (Dalman) (Hymenoptera: Chalcididae). The average parasitism rate of *A. pullata* by parasitoids was as high as 11.0%. *V. turrita* was the dominant species, attacking 10.0% of the *A. pullata* cocoons. The emergence peak of *V. turrita* was from late May to early June. Three hyperparasitoids of *V. turrita* emerged from cocoons of *A. pullata*: *Mesochorus ichneutese* Uchida (Hymenoptera: Ichneumonidae), *Pediobius* sp. (Hymenoptera: Eulophidae), and *Taeniogonalos maga* (Teranishi) (Hymenoptera: Trigonalidae). Hyperparasitism rates were about 1.0% to 3.0%, with an average rate of 1.7%.

## Introduction

The argid sawfly, *Arge pullata* (Zaddach) (Hymenoptera: Argidae), is an injurious leaf feeder of birch, *Betula* spp., in China, Europe, Siberia, and Japan (Escherich 1942; Takizawa 1962; Austara et al. 1984; Li and Yuan 1993; Zhelochovtsev and Zinovjev 1995; Nuorteva and Nuorteva 2007; Hara and Shinohara 2008; Min et al. 2010). In China, this sawfly was first recorded in Tianzhu and Yongdeng, Gansu Province, and Huzhu, Qinghai Province. Heavy infestations in birch forests were caused by the larvae in Qilian Mountains in 1991, in Huzhu County, Qinghai, in 1996 and 1997, and in Shennongjia Forest, Hubei, in 2008 and 2009 (Li and Yuan 1993; Qi 2000; Min et al. 2010). In addition to the damage to birch, the larvae of *A. pullata* have a toxin that, if ingested, causes poisoning of sheep, cattle, and goats in Denmark (Brummerstedt et al. 1987; Thamsborg et al. 1987).

The biology, occurrence, and integrated management of *A. pullata* have been studied less in China (Li and Yuan 1993; Qi 2000) than elsewhere. In Japan, this sawfly was first recorded in Sugadaira, Nagano Prefecture, central Honshu, where it had a univoltine life cycle (Takizawa 1962). Hara and Shinohara (2008) reviewed the taxonomy, distribution, life history, and economic importance of *A. pullata* in Sapporo, Hokkaido, where *A. pullata* probably had two generations a year. In China, *A. pullata* had a univoltine life cycle (Li and Yuan 1993; Qi 2000).

High population levels of sawflies are not only affected by natural collapse, but are regulated by natural enemies (Luo et al. 2005). In China, most studies on parasitoids of sawflies have involved Diprionidae, Pamphiliidae, and some Tenthredinidae that damage forest trees (Wang et al. 1996; Zhang and Zhou 1996; Wang et al. 2000; Sheng and Chen 2001; Sheng et al. 2002; Chen and Sheng 2007). Worldwide, little is known about parasitism of Argidae, although they support a parasitoid complex that is highly distinctive (Pschorn-Walcher and Kriegl 1965).

Parasitoids play an important role in biological control of agriculture and forest pests. Up until now, only two parasitoids, *Endasys parviventiris nipponicus* (Uchida) and *Mastrus nigrus* Sheng (Hymenoptera: Ichneumonidae), have been reported on *A. pullata* (Min et al. 2010; Sheng and Zeng 2010). The data on additional parasitoids presented here are important for understanding the natural control of *A. pullata*, and future biological control effect against this economically important sawfly.

## Materials and Methods

Overwintering cocoons of *A. pullata* under the bark of *Betula* spp. were collected on four occasions at the same site in Shennongjia National Nature Reserve, Hubei Province, in October 2009 and April 2010. The first sample (N = 2971) was collected on 8 October 2009, whereas the additional three samples (N = 4497, 12082 and 7075) were taken on 26 April 2010. The cocoons were reared in the laboratory at room temperature, and misted with distilled water one to two times per week in order to prevent desiccation. After one week, cocoons were stored individually in glass tubes (100 mm long and 15 mm in diameter) with a piece of filter paper dipped in distilled water (to prevent desiccation), and plugged with absorbent cotton.

All cocoons were checked daily for sawfly and parasitoid emergence until late autumn. Emerged parasitoid larvae and pupae were kept in glass tubes at room temperature until adult emergence. After emergence of sawflies and parasitoids was complete, all remaining cocoons were dissected, and their condition (i.e., status of argid sawfly, and parasitism) was recorded.

Tachinid parasitoids were identified by Dr. Chun-Tian Zhang (Shenyang Normal University), chalcid parasitoids were identified by Dr. Yan-Zhou Zhang (Institute of Zoology, Chinese Academy of Sciences), trigonalid wasps were identified by Dr. David R. Smith (Department of Entomology, National Museum of Natural History, Smithsonian Institution, United States of America). All specimens were deposited in the Insect Museum, General Station of Forest Pest Management, State Forestry Administration, P. R. China.

Parasitism rates data (*p*) were transformed by arcsin(*p*)^½^ in order to better fit the assumptions of normality and homogeneity of variances for ANOVA. The means were analyzed by one-way ANOVA, followed by the Ryan-Einot-Gabriel-Welsh (REGW) multiple Q test (SPSS 13.0 for Windows) at α = 0.05.

## Results

### Parasitoids

Five parasitoid species were reared from *A. pullata*: *Pleolophus suigensis* (Uchida), *Mastrus nigrus* Sheng, *Endasys parviventris nipponicus* (Uchida) (Hymenoptera: Ichneumonidae), *Vibrissina turrita* (Meigen) (Diptera: Tachinidae), and *Conura xanthostigma* (Dalman) (Hymenoptera: Chalcididae). The overall parasitism rates of *A. pullata* larvae by these parasitoids ranged from 8.1% to 13.8%, with an average rate of 11.0% (Table 1).

Only seven adults of the three parasitic ichneumonids, *P. suigensis*, *M. nigrus*, and *E. parviventris nipponicus* (Hymenoptera: Ichneumonidae), emerged from the cocoons of *A. pullata*, and 0, 28, 26 and 25 larvae were discovered from dissecting host larvae of the samples 1–4, respectively. The larval ichneumonids were indistinguishable from each other, so their parasitism rates were pooled and discussed as a group. Parasitism rates of *A. pullata* cocoons by ichneumonids were from 0.1% to 0.6%, with an average rate of 0.3%. Ichneumonids pupated inside the sawfly cocoons after feeding on the sawfly larva. Adults made an irregular emergence hole in the sawfly cocoons when emerging.

*V. turrita* larvae developed in the body of final instar *A. pullata* larvae, and killed them after they emerged from hosts. The tachinid final instar larvae pupated inside or outside the sawfly cocoon until adult emergence. Emergence holes were observed on the sawfly cocoons. One dead tachinid pupa was found inside the final instar larva of *A. pullata* when dissected. There were 401, 245, 694, and 535 adults tachinids that emerged from the cocoons of *A. pullata* in each sample, respectively. Parasitism rates of *A. pullata* larvae by *V. turrita* were from 7.0% to 13.5%, with an average rate of 10.0%.

*C. xanthostigma* larvae developed in the body of final instar *A. pullata* larvae, killed them after they emerged from hosts, and pupated inside the sawfly cocoon until adult emergence, leaving their puparium in the sawfly cocoon. Parasitism rates of *A. pullata* larvae by *C. xanthostigma* were from 0.1% to 1.6%, with an average rate of 0.6%.

**Figure 1.**
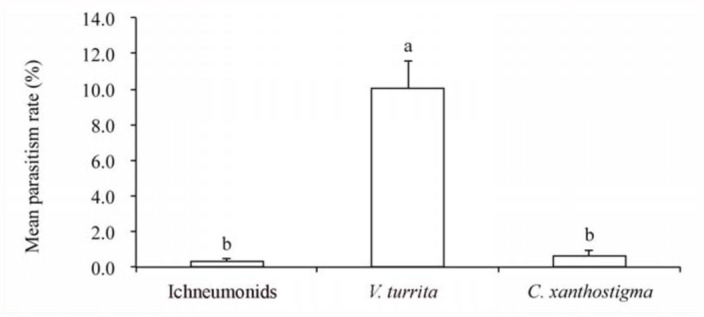
Mean parasitism rates (+ SE, n = 4) of Arge *pullata* by parasitoids in Shengnongjia. Bars with the same letter are not significantly different (ANOVA on arcsin (p)½, followed by the Ryan-Einot-Gabriel-Welsh (REGW) multiple Q test, at : α = 0.05). High quality figures are available online.

There were significant differences in the parasitism rates among the three groups of parasitoids (F = 54.97; df = 2, 9; *p* < 0.001). *V. turrita* was the dominant parasitoid, with its parasitism rates being significantly higher than those of both ichneumonids and *C. xanthostigma* (*p* < 0.001). There were no significant differences in the parasitism rates between the latter two parasitoid groups (*p* > 0.05) (Figure 1).

**Figure 2.**
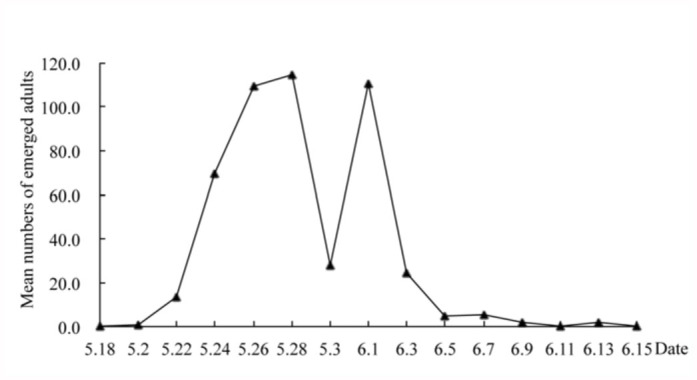
Seasonal occurrence of *Vibrissina turrita* adults emerging from final instar larvae of Arge *pullata* in 2010. High quality figures are available online.

The seasonal occurrence of *V. turrita* adults emerging from final instar larvae of *A. pullata* was recorded in 2010 (Figure 2). The emergence of *V. turrita* had two peaks. The first peak occurred from 22–28 May 2010, with 114 flies emerging on 28 May, 2010. The second peak was on 1 June 2010, with 111 flies emerging. The number of *V. turrita* emerging dropped gradually from 3 June 2010, and ended on 15 June 2010. Overall, the emergence peaks of *V. turrita* were from late May to early June.

### Hyperparasitoids

Three species of hyperparasitoids, *Mesochorus* ichneutese Uchida (Hymenoptera: Ichneumonidae), a species of *Pediobius* sp. (Hymenoptera: Eulophidae), and *Taeniogonalos maga* (Hymenoptera: Trigonalidae), were reared from *V. turrita*. Hyperparasitism rates were from 1.0% to 2.8%, with the average of 1.7%. *M. ichneutese* was only found in the 4th sample of *A. pullata* cocoons, with a hyperparasitism rate of 1.1%. One to two *Pediobius* sp. emergence holes were found on the puparium of *V. turrita*, and its hyperparasitism rates ranged from 0.2% to 0.7%, with an average rate of 0.5%. An irregular emergence hole on the pupae of *V. turrita* was characteristic of *T. maga*. Its hyperparasitism rates were from 0.2% to 1.6%, with an average rate of 1.0% (Table 2).

**Table 1.**
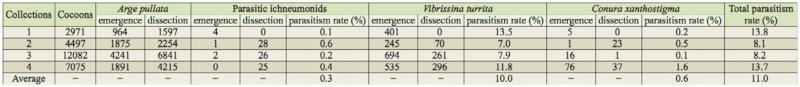
Parasitism rates (%) of Arge *pullata* cocoons by parasitoids in Shennongjia, Hubei Province.

**Table 2.**
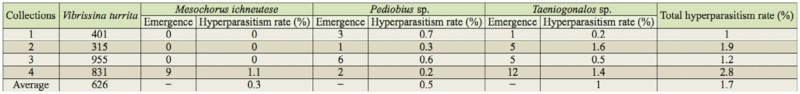
Hyperparasitoids and their hyperparasitism rates (%) of *Vibrissina turrita* puparia reared from Arge *pullata* cocoons.

## Discussion

Under the experimental conditions, the average parasitism rate of *A. pullata* by parasitoids was as high as 11.0%. There may be a difference in parasitism rate between our experiment and field conditions. Natural enemies can continuously parasitize sawflies, so the parasitism rate for *A. pullata* sawflies in nature may be higher than it was in experimental conditions. Parasitoids play an important control effect on *A. pullata* populations in nature.

*V. turrita* was the dominant parasitoid, attacking 10.0% of *A. pullata* larvae, and it plays an important role in controlling these pests. *V. turrita* has also been recorded as a parasite on other sawflies, such as *Allantus luctifer* (Smith), *Craesus varus* (Villaret), *Eriocampa ovata* (L.), *Empria abdominalis* (Fabricius), *Athalia rosae ruflcornis* Jakovlev, *Macrophya albicincta* (Schrank), *Pristiphora erichsonii* (Hartig), *Arge ustulata* (L.), *A. enodis* (L.), *A. pagana* (Panzer), and *A. ochropus* (Gmelin) (Herting 1960; Shima 1983; Nagasaka 1988; Campadelli 1997). Most of *V. turrita* adults emerged from late May to early June, so timing was an important factor when using this parasitoid to control *A. pullata*.

Most Trigonalidae are recorded as hyperparasitoids of endoparasitic ichneumonids and tachinids, or parasitoids of vespids (Yamane and Yamane 1975; Gelhaus 1987; Smith 1996; Smith and Stocks 2005). Some trigonalids are parasitoids of sawflies, such as *Taeniogonalos venatoria* Riek on *Perga affinis affinis* Kirby (Raff 1934; Carne 1969). The biology and host relationships of trigonalid wasps were studied by Clausen (1931), Carmean (1991), Weinstein and Austin (1991), and Smith (1996).

Hyperparasitism rates of *Compsilura concinnata* (Meigen) by trigonalid wasps were from 16.0% to 47.0% (Kellogg et al. 2003). Hyperparasitism rates of *V. turrita* by *T. maga* were from 0.2% to 1.6%. The biology and host relationships of *T. maga* need more investigation.

The populations and effectiveness in biological control of parasitoids are sometimes restrained by hyperparasitoids. *Marietta carnest* (Howard) and *Ablerus perspeciosus* Girault are hyperparasitoids of *Coccobius azumai* Tachikawa, which was introduced from Japan to China (Huang 1994). Hyperparasitism can be as high as 16.0% in nature (Huang et al. 2005). The parasitoid *Aphidius gifuensis* Ashmead is a very important agent in biological control efforts against of *Myzus persicae* (Sulzer). However, hyperparasitoids have one of the main restriction factors in *M. persicae* control (Ren et al. 2000).

In biological control, introduced natural enemies may sometimes compete with native natural enemies, and weaken their overall control effect on target pests. Thus, future studies on host-parasite-hyperparasite interactions, and relations between foreign and native natural enemies, would provide valuable information needed for a successful biological control program against target pest insects, including the argid sawfly, *A. pullata*.
